# In vitro evaluation of the α-glucosidase inhibitory potential of methanolic extracts of traditionally used antidiabetic plants

**DOI:** 10.1186/s12906-019-2482-z

**Published:** 2019-03-25

**Authors:** Astha Bhatia, Balbir Singh, Rohit Arora, Saroj Arora

**Affiliations:** 1 Department of Botanical and Environmental Sciences, Guru Nanak Dev University, Amritsar, Punjab 143005 India; 2 Department of Pharmaceutical Sciences, Guru Nanak Dev University, Amritsar, Punjab 143005 India; 3 Department of Biochemistry, Sri Guru Ram Das University of Health Sciences, Amritsar, Punjab 143501 India

**Keywords:** α-Glucosidase, *Clematis grata* wall., *Cornus capitata* wall., *Roylea cinerea* (D. Don) Baill

## Abstract

**Background:**

Different plant parts of *Roylea cinerea* (D. Don) Baill. (Lamiaceae), *Clematis grata* Wall. (Ranunculaceae), *Cornus capitata* Wall. (Cornaceae) are traditionally used in the management of diabetes and various other diseases.

**Method:**

The air-dried plant parts from different plants were coarsely powdered and macerated in methanol to obtain their crude extracts. The crude extracts were evaluated for their α-glucosidase inhibitory activity. On the basis of results obtained, the methanolic crude extract of *Cornus capitata* Wall. was further sequentially fractionated in hexane, diethyl ether, ethyl acetate, n-butanol. Fractions obtained were also evaluated for their α-glucosidase inhibitory potential. The kinetic study was performed using Lineweaver Burk plot to evaluate the type of inhibition. Furthermore, in silico analysis was also carried with active sites of the enzyme (PDB ID: 3WY1) using Autodock4.

**Results:**

Among all the plant extracts, *Cornus capitata* extract showed maximum inhibitory activity. Therefore its methanolic extract was further fractionated with the help of different solvents and the maximum activity was shown by the ethyl acetate fraction (IC_50_ 50 μg/mL). Kinetic analysis indicated that Vmax and Km were increased indicating a competitive type of inhibition. In docking studies, among different constituents known in this plant, betulinic acid showed minimum binding energy (− 10.21 kcal/mol). The kinetic and docking studies have strengthened the observation made in the present study regarding the α-glucosidase inhibitory activity of *Cornus capitata*.

**Conclusion:**

The study provided partial evidence for pharmacological basis regarding clinical applications of *Cornus capitata* in the treatment of diabetes suggesting it to be a suitable candidate for the treatment of postprandial hyperglycemia.

**Electronic supplementary material:**

The online version of this article (10.1186/s12906-019-2482-z) contains supplementary material, which is available to authorized users.

## Background

Diabetes is the world’s fastest-growing metabolic endocrine disorder with compromised carbohydrate and lipid metabolism. The defected metabolism can be attributed to the impaired insulin secretion, insulin action or both. Alpha-glucosidase inhibitors are one of the effective class of antidiabetic therapeutics capable of ameliorating hyper-glycemia especially postprandial hyperglycemia over alpha-amylase inhibitors [1]. Postprandial hyperglycemia is a sudden elevation in glucose levels in the blood after a meal. Major constituents of the human diet are comprised of carbohydrates such as starch and sucrose. The membrane-bound α-glucosidase enzyme, localized in the epithelium of small intestine hastens the digestion of oligosaccharides and disaccharides into simple glucose, after which it gets absorbed and enter into the bloodstream [2]. Inhibition of α-glucosidase enzyme can help in delaying digestion of carbohydrates, thereby reducing the levels of glucose in blood [3]. Several drugs are available as α-glucosidase inhibitors such as acarbose and miglitol that inhibit the absorption of carbohydrates from the gut. These inhibitors can prevent or mask the manifestation of the disease. There are several studies reported for such inhibitors being beneficial to prevent or delay impaired glucose tolerances in diabetes. Though being effective, these drugs have been reported to cause severe side effects such as gastrointestinal discomforts including diarrhea and flatulence [4].

Natural products of plant origin are known for their dual role both as preventive and therapeutic agents, combined with their less toxicity and side effects. Traditionally different plants are used throughout the world by the local population for the treatment of various ailments [5]. *Cornus capitata* Wall. (Cornaceae) is widely available in different regions of the world such as China and India. Studies have shown that it has several constituents present in its aerial parts such as cornin and phlorin from leaves, others include n-hentriacontane, 7-hydrocadeline, triacontanol, stigmastranone, lupeol, triacontanoic acid, betulin, epibetulin, betulinic acid, epibetulinic acid, tetracosanoic acid, maslinic acid, arjunolic acid, sitosterol-β-D-glucoside, gallic acid isolated from stem [6, 7]. It is used as fuel, for animal bedding and its fruits are edible [8]. It has been reported for its traditional use in the management of diabetes in Mandi district of Himachal Pradesh [9]. *Clematis grata* Wall. (Ranunculaceae), is used by indigenous people of Bana valley (Jammu and Kashmir) for its antioxidant, antimicrobial, and antidiabetic activities [10]. Its crushed leaves are used as a paste for the treatment of skin diseases such as eczema, warts, carbuncles [11] and boils [9]. Two important constituent viz., clematoside-S and triterpenoids saponins have been reported from the roots of *Clematis grata* Wall. [12]. *Roylea cinerea* (D. Don) Baill. (Lamiaceae) found in Western Himalayas had been used by the local population in the management of various diseases including diabetes, fever, skin diseases [9, 13]. Rutin, isoquercetin, nicotiflorin and martynoside are some constituents reported from its aerial parts [13]. Thus, the present study was conducted with an objective to unravel the α-glucosidase inhibitory effect of *Cornus capitata* Wall. (leaves), *Clematis grata* Wall. (whole plant)*, Roylea cinerea* (D. Don) Baill.(leaves and stem) based on their traditional use for the management of diabetes [9, 10]. Kinetic study and in silico analysis was also carried out to further investigate the mechanism of inhibition.

## Methods

### Plant extraction

The entire plant or its different parts can be used in the form of a whole plant, crude extract, and aqueous decoctions. In the present study, the whole plant was selected in case of herbs, leaves and stem in case of shrubs and leaves in case of trees on the basis of availability of phytoconstituents [14]. Plant materials viz., (*Cornus capitata* Wall. (leaves), *Clematis grata* Wall. (Whole plant), *Roylea cinerea* (D. Don) Baill. (Leaves and stem) were collected, identified and submitted as a voucher specimen (Accession no. 7373, 7374 and 7376 respectively) for authenticity in Herbarium, Department of Botanical and Environmental Sciences, Guru Nanak Dev University, Amritsar, Punjab (Additional file 1: Table S1). The identification was done by taxonomist Dr. Amarjit Singh Soodan and Mr. Ram Prasad (Senior lab technician), of Department of Botanical and Environmental Sciences, Guru Nanak Dev University, Amritsar, Punjab. Each plant material was air dried, coarsely powdered and subjected to maceration for 48 h in methanol. The methanolic extracts were further concentrated with a rotary evaporator (IKA® RV 10). The extracts were stored at − 20 °C until use. Crude methanolic extract was dissolved in distilled water. Further, an equal volume of Hexane was added and the mixture was shaken vigorously. The mixture was allowed to separate into layers. The upper layer (Hexane) was collected and concentrated on rotary evaporator to obtain Hexane fraction. Similarly, the aqueous layer was further subjected to fractionation via series of solvents on the basis of polarity viz., Diethyl-ether<Ethyl acetate<n-butanol. Polar solvents help in the extraction of hydrophilic compounds and non-polar solvents aids in the extraction of lipophilic compounds [15, 16].

### α-Glucosidase inhibitory assay

Plant extracts were evaluated for α-glucosidase inhibitory activity according to the method given by Pistia Brueggeman and Hollingsworth, 2001 [17] with slight modifications. Plant extracts (50 μL) at varying concentrations (12.5 to 400 μg/mL) was incubated with 10 μL of the α-glucosidase (maltase) ex. Yeast (Sisco Research Laboratories Pvt. Ltd.) enzyme solution (1 U/mL) for 20 min at 37 °C with an additional 125 μL of 0.1 M phosphate buffer (pH 6.8). After 20 min, the reaction was started with the addition of 20 μL of 1 M pNPG (substrate) and the mixture was incubated for 30 min. The reaction was terminated with the addition of 0.1 N of Na_2_CO_3_ (50 μL) and final absorbance was measured at 405 nm using Biotek multi-well plate reader. Acarbose was used as a positive control at varying concentrations (12.5 to 400 μg/mL). Enzyme activity was calculated as:

### (OD_BLANK_-OD_SAMPLE_)/OD_BLANK_ x 100

One unit of the enzyme can be defined as the amount of enzyme (α-glucosidase) required for the formation of one μmol of product (p-Nitrophenol) from the substrate (*p*-nitrophenyl-*α*-D-glucopyranoside) per minute. IC_50_ (concentration required to inhibit 50% of the enzyme activity) was calculated using regression equation obtained through plotting concentration in the range 12.5–400 μg/mL (x-axis) and %inhibition (y-axis) for different extracts and fractions.

### Kinetic study

Lineweaver-Burk plot was plotted for methanolic extract of *Cornus capitata* Wall. with IC_50_ of 12.45 μg/mL. The substrate p-nitrophenyl-α-D-glucoside was used in varying concentrations (2 mM, 1 mM, 0.5 mM, 0.25 mM and 0.125 mM). Enzyme concentration was kept constant (1 U/mL) and molar extinction coefficient for calculating enzyme activity was 17.4 mM^− 1^ [3]. Inhibition type was determined with Lineweaver-Burk plots and calculated using the Michaelis Menten equation.

1 $$ Vo=\frac{Vmax\left[S\right]}{Km+\left[S\right]} $$

Rearranging the equation for Line-weaver Burk plot (linear, y = ax+b).

2 $$ \frac{1}{Vo}=\frac{Km+\left[S\right]}{Vmax\left[S\right]}=\frac{Km}{Vmax}\frac{1}{\left[S\right]}+\frac{1}{Vmax} $$

Where V_0_ is the initial rate of reaction, Km is the Michaelis Menten constant, Km/Vmax is the slope, and the y-intercept (1/Vmax) was calculated using Eq. 2.

### Molecular docking

**Fig. 1:**
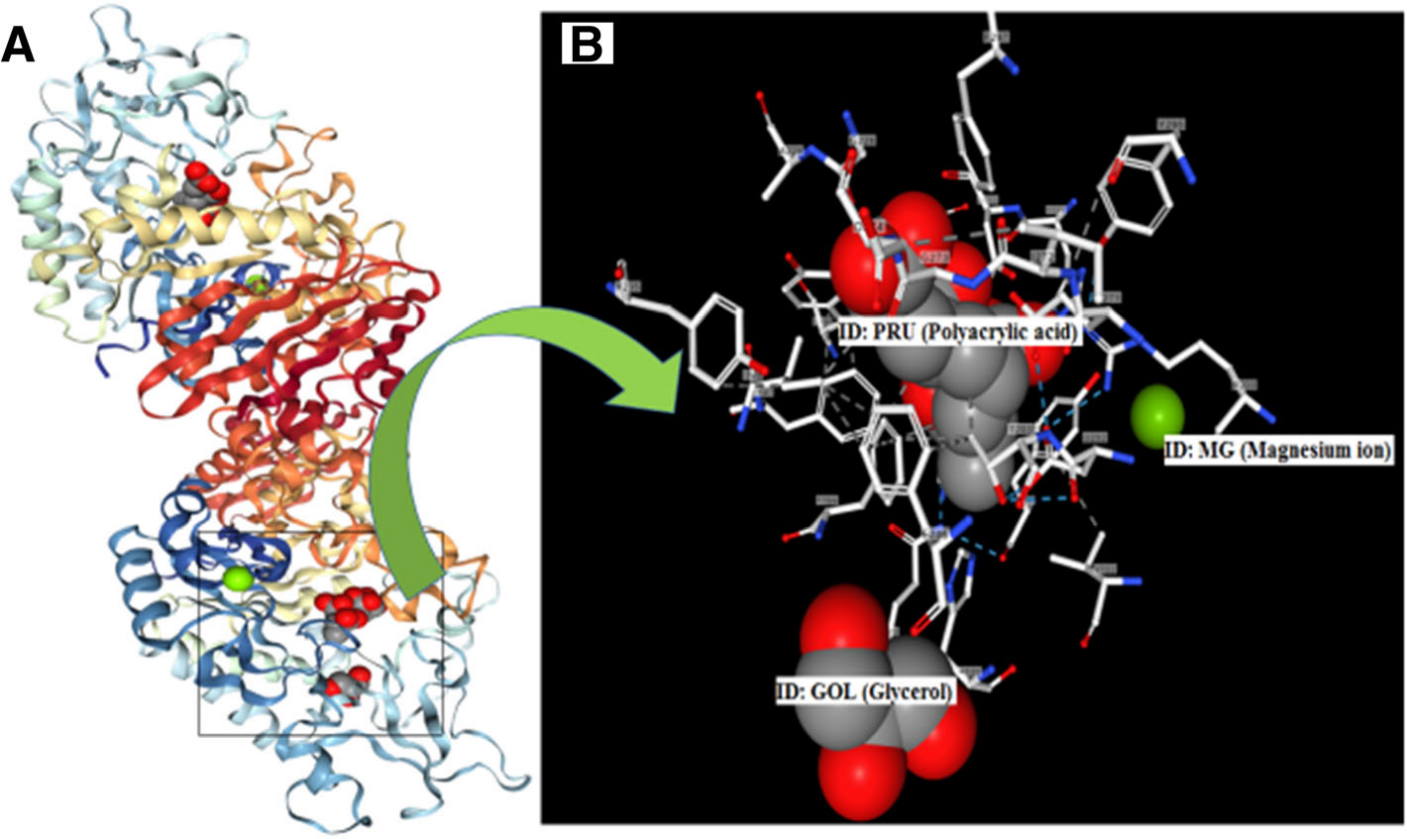
Protein structure of homologue of α-glucosidase obtained from RCSB protein data bank for docking **a**. Structure of 3WY1 protein (chain **a** and **b**); **b**. Binding pocket showing binding with ligands

The methanolic extract of *Cornus capitata* Wall. exhibited maximum α-glucosidase inhibitory activity, therefore the docking studies were carried out for the chemical constituents already reported in this plant. The chemical structure and molecules for which docking was carried out are mentioned in Additional file 1: Table S2. In this study, the protein structure of the target enzyme α-glucosidase (3WY1) and a binding pocket for polyacrylic acid was obtained from the protein data bank (www.rcsb.org) (Fig. 1). The 3WY1 protein consists of 3 ligands namely polyacrylic acid (ID: PRU), glycerol (ID: GOL) and magnesium ion (ID: MG). The preparation of the target enzyme with the AutoDock4 involved the addition of hydrogen atoms to the target enzyme, which is a necessary step for the computation of partial atomic charge. Hetero-atoms present in the protein structure 3WY1 were removed prior to autodock analysis. Gasteiger charges were considered for each atom present in the target. It executes automated docking of user-specified ligands into protein binding pocket. Three-dimensional affinity grid size − 10.620, − 15.320, 17.310 (x, y, and z) on the geometric center of the target protein was used. Docking algorithm was run using Cygwin software, to obtain the binding energy data. Visualization and analysis of the results were done using UCSF chimera 1.11rc. Ligand structures were obtained from PubChem (https://pubchem.ncbi.nlm.nih.gov/search/search.cgi) and prepared using chemsketch tool.

### Statistical analysis

The data for inhibitory activity of plant extracts and their formulations for α-glucosidase enzyme are presented as mean ± standard error. The data was further analyzed using one-way analysis of variances (ANOVA) and Tukey’s test for multiple comparison tests assuming variances are equal using IBM SPSS version 16.0 software.

## Results

### In vitro α-glucosidase inhibitory activity

**Fig. 2:**
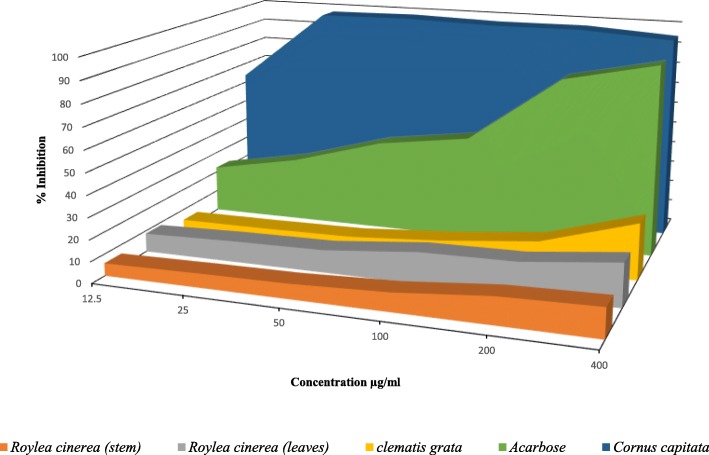
Comparative representation of α-glucosidase inhibitory activity (% Inhibition by plant extracts and standard Acarbose)

Crude methanolic extracts of (*Cornus capitata* Wall. (leaves), *Clematis grata* Wall. (Whole plant) and *Roylea cinerea* (D. Don) Baill. (leaves and stem) were evaluated for their α-glucosidase inhibitory activity. The methanolic extract of *Cornus capitata* Wall. was sequentially extracted with different solvents to obtain fractions. It was observed that *Cornus capitata* Wall. (leaves), *Clematis grata* Wall. (Whole plant), *Roylea cinerea* (D. Don) Baill. (leaves and stem) extracts exhibited 98.37, 5.05, 10.50 and 7.01% inhibitory activity at 50 μg/mL respectively (Table 1 and Fig. 2). Among the different plants, *Cornus capitata* Wall. extract exhibited maximum inhibitory activity with 98.37% inhibition (IC_50_ 12.5 μg/mL) better than the activity shown by positive control (acarbose) with 42.43% inhibition (IC_50_ 66.07 μg/mL) at 50 μg/mL concentration. The fractions viz., hexane, diethyl ether, ethyl acetate and n-butanol obtained from an extract of *Cornus capitata* Wall. exhibited 98.98, 96.54, 98.55, 98.35% inhibitory activity at 200 μg/ml respectively (Table 2). It was observed that the inhibitory activity for α-glucosidase enzyme was maximum by ethyl acetate (IC_50_ 50 μg/ml) followed by n-butanol (IC_50_ 61.175 μg/mL), hexane (IC_50_ 65.12 μg/mL) and diethyl ether fractions (IC_50_ 71.85 μg/mL).

**Table 1:** α-glucosidase activity of methanolic extracts of different medicinal plants and Acarbose (standard drug)

Plant	Percentage α-glucosidase inhibition (%)						IC 50 (μg/mL)
	12.5 μg/mL	25 μg/mL	50 μg/mL	100 μg/mL	200 μg/mL	400 μg/mL	
Acarbose (Standard)	18.83 ± 3.87^b^	30.18 ± 0.06^b^	42.43 ± 0.05^b^	56.31 ± 0.02^b^	80.10 ± 0.15^b^	89.40 ± 0.09^a^	66.07
*Cornus capitata* (Leaves)	63.68 ± 1.98^a^	97.81 ± 0.03^a^	98.37 ± 0.12^a^	97.43 ± 0.18^a^	97.18 ± 0.18^a^	94.70 ± 0.41^a^	12.5
*Clematis grata* (Whole plant)	0.32 ± 5.79^b^	4.83 ± 1.46^c^	5.05 ± 0.74^d^	5.65 ± 0.75^b^	8.72 ± 5.09^c^	13.25 ± 1.10^b^	**–**
*Roylea cinerea* (Leaves)	8.66 ± 0.20^b^	9.87 ± 0.16^c^	10.50 ± 0.66^c^	14.62 ± 4.20^b^	15.31 ± 2.60^c^	20.09 ± 2.21^b^	**–**
*Roylea cinerea (Stem)*	5.85 ± 1.50^b^	6.54 ± 3.04^c^	7.01 ± 1.23^d^	8.69 ± 1.87^b^	12.57 ± 2.97^c^	13.61 ± 2.10^b^	**–**

Values are mean ± S.E. of three parallel measurements

Different letters indicate significant differences between same concentrations of different extracts and acarbose (*p* < 0.05, Tukeys HSD test, F-ratio- 63.89 (12.5 μg/mL), 687.88 (25 μg/mL), 3.24E3 (50 μg/mL), 13.29 (100 μg/mL), 212.97 (200 μg/mL), 291.66 (400 μg/mL)

*Cornus capitata* (4.25% at 1.5 μg/mL; 6% at 3.1 μg/mL and 19% inhibition at 6.25 μg/mL concentration)

**Table 2:** α-glucosidase activity of methanolic extract of *Cornus capitata* and its fractions

Plant extract/Fractionate	Percentage α-glucosidase inhibition						IC 50 (μg/mL)
	12.5 μg/mL	25 μg/mL	50 μg/mL	100 μg/mL	200 μg/mL	400 μg/mL	
*Cornus capitata* (Leaves)	63.68 ± 1.98^a^	97.81 ± 0.03^a^	98.37 ± 0.12^a^	97.43 ± 0.18^a^	97.18 ± 0.18	94.70 ± 0.41	12.5
Hexane Fraction	2.78 ± 2.73^b^	16.04 ± 4.78^b^	31.94 ± 5.17^b^	69.07 ± 2.51^a^	98.98 ± 1.22	96.52 ± 3.02	65.12
Diethyl ether Fraction	8.31 ± 0.87^b^	13.04 ± 2.80^b^	30.98 ± 4.21^b^	52.64 ± 6.11^a^	96.54 ± 0.92	95.65 ± 0.65	71.85
Ethyl acetate Fraction	14.60 ± 3.07^bc^	18.42 ± 2.65^b^	50.06 ± 0.36^bc^	84.45 ± 2.80^a^	98.55 ± 0.51	97.45 ± 1.08	50
n-Butanol Fraction	10.57 ± 0.21^bc^	14.93 ± 2.83^b^	26.07 ± 4.53^bc^	78.75 ± 4.63^ab^	98.35 ± 0.24	97.75 ± 0.43	61.175

Values are mean ± S.E. of three parallel measurements

Different letters indicate significant differences between same concentrations of *Cornus capitata* methanolic extract and its fractionates (p < 0.05, Tukeys HSD test, F-ratio- 49.935 (12.5 μg/mL), 55.279 (25 μg/mL), 46.702 (50 μg/mL), 16.690 (100 μg/mL), 1.861 (200 μg/mL), 0.683 (400 μg/mL)

*Cornus capitata* methanolic extract (leaves) (4.25% at 1.5 μg/mL; 6% at 3.1 μg/mL and 19% inhibition at 6.25 μg/mL concentration)

### Kinetic study

**Fig. 3:**
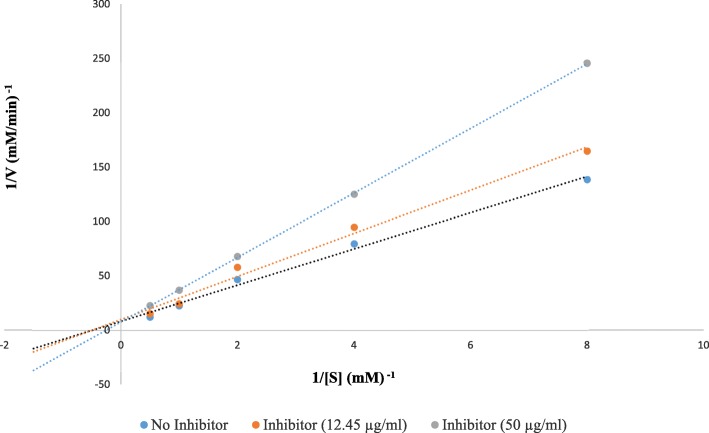
Lineweaver-Burk plot of the reaction of yeast α-glucosidase in the presence of inhibitor methanolic extract of *Cornus capitata* leaves extract

Enzyme kinetic study was carried out for the investigation of the type of inhibition by the crude methanolic extract of *Cornus capitata* Wall. The Lineweaver-Burk plot (Fig. 3) revealed that the extract showed a competitive type of inhibition. The y-intercept (1/Vmax) remains unaffected and slope (Km/Vmax) increased with the concentration of the inhibitor. The value of α, the degree of inhibition (constant for the degree of inhibition affecting the affinity of the enzyme to the substrate) was greater than 1. This confirms that the inhibitor preferentially binds to the free enzyme inhibiting the formation of the enzyme-substrate complex.

### Molecular docking

**Fig. 4:**
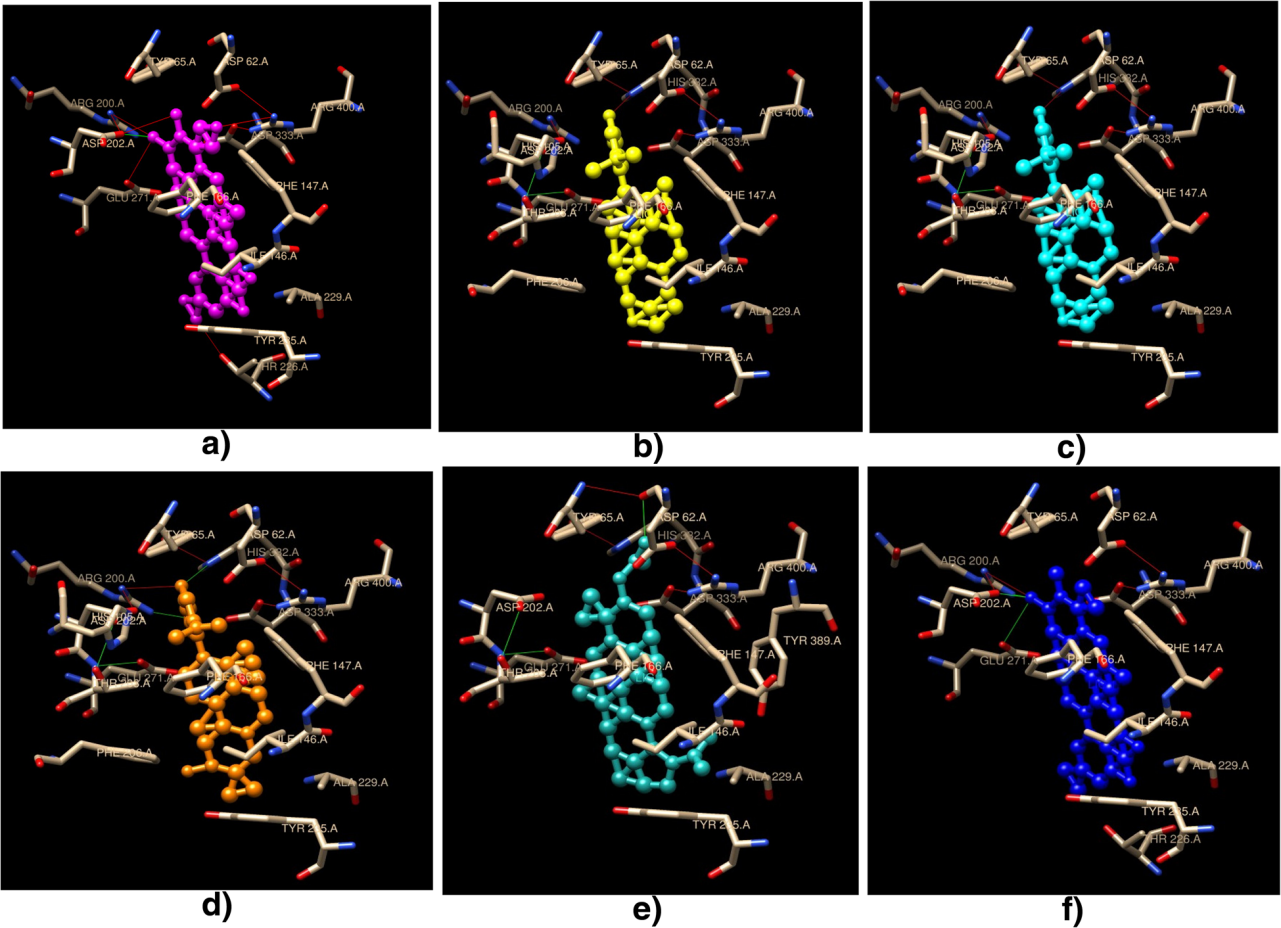
Docking conformations (Chimaera software) showing interaction (residue-ligand H-bonds green coloured) of **a**) Arjunolic acid **b**) Betulin **c)** Betulinic acid **d**) Epibetulin **e**) Lupeol **f**) Maslinic acid with binding site of α-glucosidase

As the methanolic extract of *Cornus capitata* Wall. showed maximum inhibition potential against α-glucosidase enzyme, docking studies were performed to investigate the activity of some of its already reported chemical constituents (betulinic acid, epibetulin, arjunolic acid, lupeol, maslinic acid, and betulin) [6, 7]. Ligands were prepared using Chemsketch software, docked into the binding site of the 3WY1 protein (α-glucosidase enzyme) and found to fit well in the binding cavity of 3WY1 protein. Autodock 4 software was used to dock the ligands into the binding pocket. Docking conformations were visualized and analyzed for the number of H-bonds formed using Chimera software (Fig. 4). It revealed that the betulinic acid, epibetulin, arjunolic acid, betulin, lupeol, and maslinic acid possess good binding potential toward α-glucosidase enzyme (Table 3). Docking conformations analyzed for each ligand explicated the interactions through the formation of H-bonds with different amino acids residues of the protein 3WY1. Betulinic acid showed no H-bonds with amino acid residues but fits well in the binding cavity with the minimum free energy of binding as − 10.21 Kcal/mol and inhibition constant of 33.04 nM. Epibetulin showed two H-bonds with amino acid residues ARG 200 and HIS 332 with bond length 2.446 Å and 2.516 Å. Arjunolic acid showed the presence of two H-bonds with amino acid residues ASP 202 and ARG 200 with a bond length of 2.287 Å and 2.726 Å, respectively. Maslinic acid showing three H-bonds with amino acid residues ASP 202, ARG 200 and GLU 271 with bond length 2.296 Å, 2.731 Å, and 3.160 Å. Lupeol showed two H-bonds with amino acid residue ASP 62 with bond length 3.101 Å and 2.472 Å.

**Table 3:** Predicted binding energies for constituents present in *Cornus capitata* docked with α-glucosidase (PDB ID: 3WY1)

S. No.	Molecules	Binding Energy (kcal/mol)	No. of H-bonds	Cluster RMSD	Reference RMSD
3	Betulinic acid	−10.21	0	0.00	25.81
4	Epibetulin	−9.06	2	0.00	26.10
1	Arjunolic acid	−6.60	2	0.00	26.51
6	Maslinic acid	−5.27	3	0.00	24.01
5	Lupeol	−4.79	2	0.00	26.16
2	Betulin	−4.00	2	0.00	25.71

## Discussion

In the present study, solvent selection for extraction purpose was done on the basis of already available literature [18]. Previous studies suggest that methanol and hot water are the ideal solvents for extraction of antioxidants and other endogenous compounds present in a plant [19]. Thus, the extraction of plant material was done by a maceration process in absolute methanol to obtain the maximum content of chemical constituents. Various studies have suggested the role of plant extracts as α-glucosidase inhibitors clearly indicating the potential of these extracts to manage hyperglycemia [20]. The activity of these plant extracts can be attributed to phytoconstituents present in them, such as flavonoids, alkaloids, terpenoids, anthocyanins, glycosides, phenolic compounds. In the present study, among all the methanolic plant extracts, *Cornus capitata* Wall. extract explicated a remarkable inhibition potential (98.37% at 50 μg/mL) against the α-glucosidase enzyme. The other methanolic extracts were found to be less effective against the α-glucosidase enzyme. On the basis of activity shown by the crude methanolic extract of *Cornus capitata* Wall., it was further fractionated successively in different polar and non-polar solvents [15]. Among its fractions, ethyl acetate fraction showed the maximum inhibitory activity. Extraction of a type of compound depends upon the selection of the solvent. Ethyl acetate fraction is known to be rich in flavonoids [17, 21]. Thus, enzyme inhibitory activity shown by ethyl acetate fraction can be attributed to its rich flavonoid contents. Critical analysis of the results obtained suggested that the methanolic crude extract of leaves of *Cornus capitata* Wall. showed better activity than its all fractionates. The decrease in the enzyme inhibitory activity shown by different fractions may be due to the synergistic effect of the chemical constituents present in the crude methanolic extract. This suggests that the activity of crude methanolic extract of *Cornus capitata* Wall. was due to the synergistic potential of chemical constituents present in it. Due to limited literature regarding the evaluation of antidiabetic activity of *Cornus capitata* Wall., the information was gathered about other species of *Cornus*. Various species of *Cornus* have been reported in the literature for showing remarkable antidiabetic activity. *Cornus officinalis* has been categorized as an important plant by the ministry of health of the People’s Republic of China and also declared as a valuable food supplement for the management of diabetes [22]. Nearly 90% of the ancient Chinese herbal medicines contain *Cornus officinalis* as an ingredient [23]. It has been reported to contain iridoid glycoside and several other constituents in its crude extract responsible for showing remarkable α-glucosidase inhibitory effects in vitro and in vivo models [24]*.* Similarly, *Cornus mas* (L.) also known as Cornelian cherry, is used in the preparation of beverages in European regions and used as an effective medicine for the treatment of Diabetes in Asia [25]. Thus, *Cornus capitata* leaves can also provide a possible medication for the better management of diabetes which has not been investigated yet.

To obtain further information regarding the type of inhibition, kinetic studies were carried out using a crude methanolic extract of *Cornus capitata* Wall. It was observed that the slope and Km increased with the increasing concentration of the extract (inhibitor) but Vmax remained unaffected which indicated that *Cornus capitata* Wall. methanolic extract inhibited the reaction to proceed through competitive interaction with the substrate. The mechanism involved in this type of inhibition includes either strong affinity or structural similarity to the substrate [25, 26]. It can facilitate its binding with the active site of the enzyme, thus inhibiting the reaction to proceed forward. Therefore, to obtain useful information about the α-glucosidase enzyme inhibitory activity by methanolic extract of *Cornus capitata* Wall., the lead regarding the chemical constituents present in it was taken from available literature [6, 7]. However, very little attention has been paid to this plant. The literature was further explored to find the evidence supporting the antidiabetic activities of the constituents present in the crude methanolic extract of *Cornus capitata* Wall. It was found that the pentacyclic triterpenoids such as arjunolic acid, betulinic acid, betulin, epibetulin, maslinic acid, and lupeol may be responsible for its antidiabetic activity [27, 28]. Consequently, in order to understand the interactions between the protein 3WY1 (α-glucosidase enzyme homolog) and the constituents present in *Cornus capitata* Wall. methanolic extract, docking studies were carried out. It revealed the significant binding efficiencies of the chemical constituents present in *Cornus capitata* Wall., with binding pocket of protein (PDB ID: 3WY1) showing the consistent result with the in vitro findings, thus strengthening the basis of the remarkable α-glucosidase inhibitory potential of *Cornus capitata* Wall. (Additional file 2: Figures S1-S7). Triterpenoids can act as lead molecules in the treatment of diabetes and its complications. Pentacyclic triterpenes such as betulinic acid, betulin, lupeol, maslinic acid etc. have been studied in both in vivo and in vitro models suggesting their potential in the treatment of diabetes and its complications [28, 29]. Stability of a ligand in the binding pocket is calculated as minimum binding energy. Betulinic acid showed minimum binding energy i.e. 10.21 Kcal/mol followed by epibetulin, arjunolic acid, maslinic acid, lupeol, and betulin. Their significant α-glucosidase inhibitory effect may be due to the main triterpene nucleus and hydroxyl groups at R1 and R2 positions (Additional file 2: Figure S8). It favors hydrogen bonding and electrostatic interactions between the binding pocket of enzyme and hydroxyl groups. Carboxyl group at R3 position favors activity potential in pentacyclic triterpenes [30]. Several studies support the potential of betulinic acid as an effective α-glucosidase inhibitor with IC_50_ varying from 7.6–14.9 μM [31, 32]. This activity can be due to hydroxyl group attached at R2 position (electron-donating group) and a carboxyl group attached. Further, replacement of hydroxyl group with carbonyl groups directly affect the inhibitory potential of pentacyclic triterpenes as in case of epibetulin. Additional hydroxyl group present other than R1 and R2 position in case of arjunolic acid decreased its inhibitory potential based on structure-activity relationship [31]. Low inhibitory potential of betulin, when compared to its derivative, betulinic acid indicated the importance of carboxylic group [33, 34]. Decreased activity of lupeol and absence of carboxylic acid in its structure can also be co-related. Maslinic acid is hydrophobic in nature with a rigid skeleton which results in its decreased bioavailability. However, its glycosylation at R2 and R3 position increase its inhibitory potential [35]. Also, molecular shape and size of a compound decide easy penetration in the binding pockets followed by its interactions. Substituent groups and their positions in case of pentacyclic triterpenoids plays a very crucial role. Thus, *Cornus capitata* Wall. can be used as an effective drug for the treatment and prevention of diabetes.

## Conclusion

In conclusion, our study demonstrated the potent α-glucosidase inhibitory potential of methanolic extract of leaves of *Cornus capitata* Wall. among different plant extracts and its fractions, suggesting that the activity was due to the synergistic potential of chemical constituents present in it. It can interfere or delay the absorption process of dietary carbohydrates leading to suppression of increase in levels of glucose after a meal. The study provided partial evidence for pharmacological basis regarding clinical applications of *Cornus capitata* in the treatment of diabetes. Further, kinetic analysis and docking studies supported the substantially strong inhibitory activity shown by the crude methanolic extract of *Cornus capitata* Wall. (leaves). However, further research including in vivo studies and isolation of the active constituents will be interesting to explore to confirm the efficacy of *Cornus capitata* Wall. leaves extract before its extensive use in suppressing hyperglycemia in diabetic patients.

## Additional files

Additional file 1: **Table S1.** Plants used in study with their parts, family with accession numbers. **Table S2.** Chemical structures of Reported chemical constituents from *Cornus capitata* prepared via Chemsketch for Docking a) Arjunolic acid b) Betulin c) Betulinic acid d) Epibetulin e) Lupeol f) Maslinic acid. (DOCX 86 kb)

Additional file 2: **Figure S1.** Docking conformation showing binding of 3WY1 protein with Betulinic acid. **Figure S2.** Docking conformation showing binding of 3WY1 protein with Epibetulin. **Figure S3.** Docking conformation showing binding of 3WY1 protein with Arjunolic acid. **Figure S4.** Docking conformation showing binding of 3WY1 protein with Maslinic acid. **Figure S5.** Docking conformation showing binding of 3WY1 protein with Lupeol. **Figure S6.** Docking conformation showing binding of 3WY1 protein with Betulin. **Figure S7.** Docking conformation showing binding of 3WY1 protein with Acarbose. **Figure S8.** Main structure of penta-cyclic triterpenoid. (DOCX 6269 kb)

## Abbreviations

ARG: Arginine
ASP: Aspartic acid
GLU: Glutamic acid
HIS: Histidine
IC_50_: 50% inhibitory concentration
PDB ID: Protein data bank ID
pNPG: *p*-nitrophenyl-*α*-D-glucopyranoside
