# Supplementary material for: In vitro evaluation of the α-glucosidase inhibitory potential of methanolic extracts of traditionally used antidiabetic plants
Source: BMC Complement Altern Med. 2019 Mar 25;19:74. doi: 10.1186/s12906-019-2482-z (PMC6434821; doi:10.1186/s12906-019-2482-z)
Supplement: Supplementary file 1 — Table S1. Plants used in study with their parts, family with accession numbers. Table S2. Chemical structures of Reported chemical constituents from Cornus capitata prepared via Chemsketch for Docking a) Arjunolic acid b) Betulin c) Betulinic acid d) Epibetulin e) Lupeol f) Maslinic acid. (DOCX 86 kb) [file 12906_2019_2482_MOESM1_ESM.docx]

**Supplementary material**

**Additional file 1: Table S1.** Plants used in study with their parts, family with accession numbers.

| **Plant (Botanical name)** | **Family** | **Collection**  **Site** | **Common name** | **Part used** | **Accession no.** |
| --- | --- | --- | --- | --- | --- |
| ***Cornus capitata* Wall.** | Cornaceae | Mandi,  Himachal Pradesh, India. | Himalayan strawberry, Evergreen dogwood | Leaves | **7373** |
| ***Clematis grata* Wall.** | Ranunculaceae | Palampur,  Himachal Pradesh, India. | Charming clematis  (Dnanwali, Garol) | Whole plant | **7374** |
| ***Roylea cinerea* (D.Don) Baill.** | Lamiaceae | Palampur,  Himachal Pradesh, India. | Ashy roylea  (Titpatti, Pitkarui) | Stem, Leaves | **7376** |

**Additional file 1: Table S2.** Chemical structures of Reported chemical constituuents from *Cornus* *capitata* prepared via Chemsketch for Docking **a)** Arjunolic acid **b)** Betulin **c)** Betulinic acid **d)** Epibetulin **e)** Lupeol **f)** Maslinic acid.

| 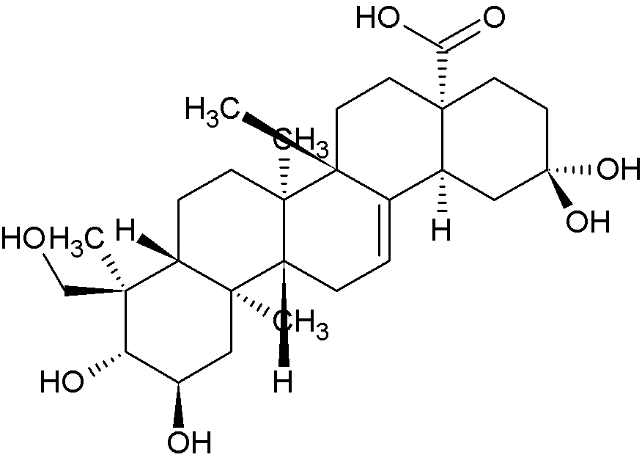  **a)** | 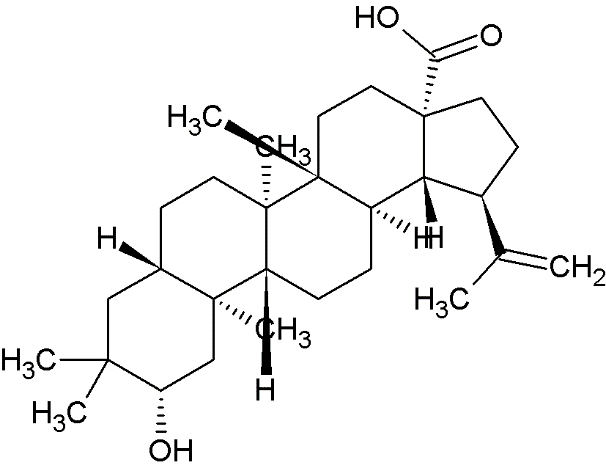  **b)** | 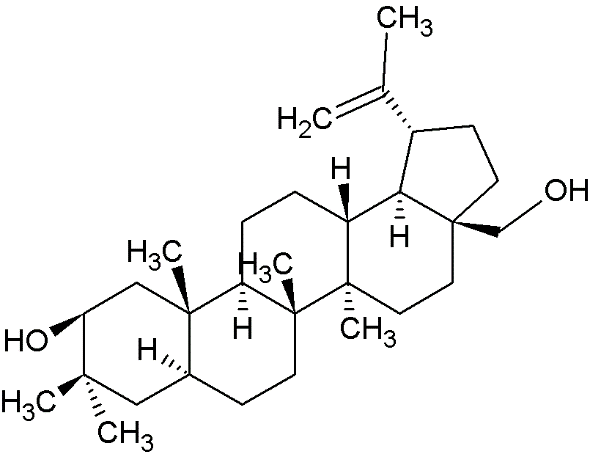  **c)** |
| --- | --- | --- |
| 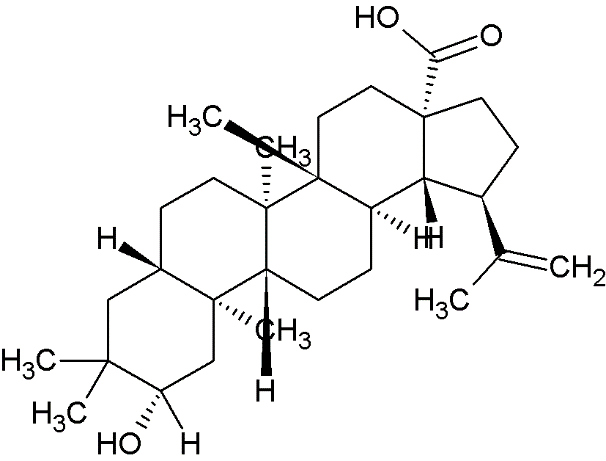  **d)** | 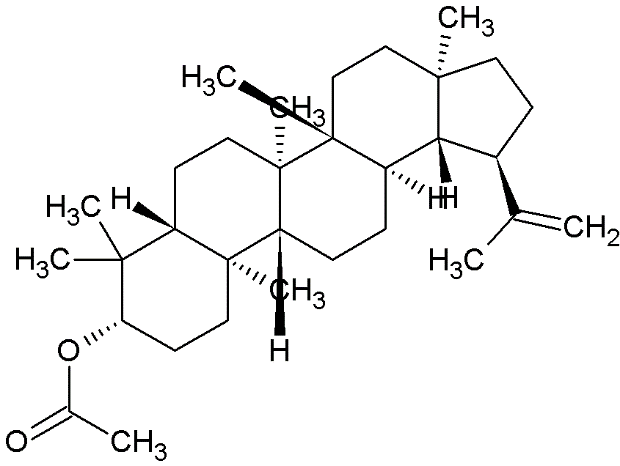  **e)** | 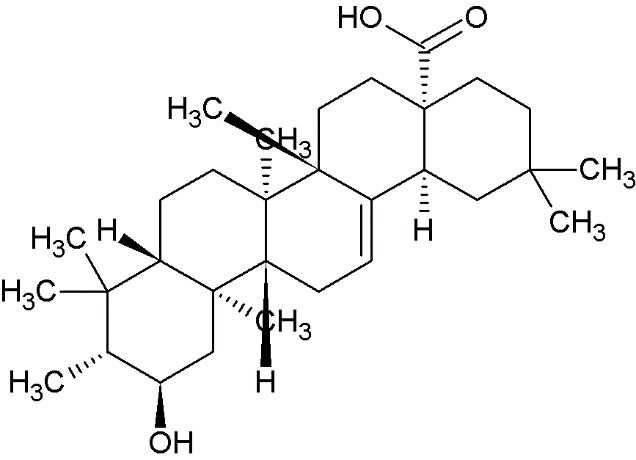  **f)** |
