# Supplementary material for: In vitro evaluation of the α-glucosidase inhibitory potential of methanolic extracts of traditionally used antidiabetic plants
Source: BMC Complement Altern Med. 2019 Mar 25;19:74. doi: 10.1186/s12906-019-2482-z (PMC6434821; doi:10.1186/s12906-019-2482-z)
Supplement: Supplementary file 2 — Figure S1. Docking conformation showing binding of 3WY1 protein with Betulinic acid. Figure S2. Docking conformation showing binding of 3WY1 protein with Epibetulin. Figure S3. Docking conformation showing binding of 3WY1 protein with Arjunolic acid. Figure S4. Docking conformation showing binding of 3WY1 protein with Maslinic acid. Figure S5. Docking conformation showing binding of 3WY1 protein with Lupeol. Figure S6. Docking conformation showing binding of 3WY1 protein with Betulin. Figure S7. Docking conformation showing binding of 3WY1 protein with Acarbose. Figure S8. Main structure of penta-cyclic triterpenoid. (DOCX 6269 kb) [file 12906_2019_2482_MOESM2_ESM.docx]

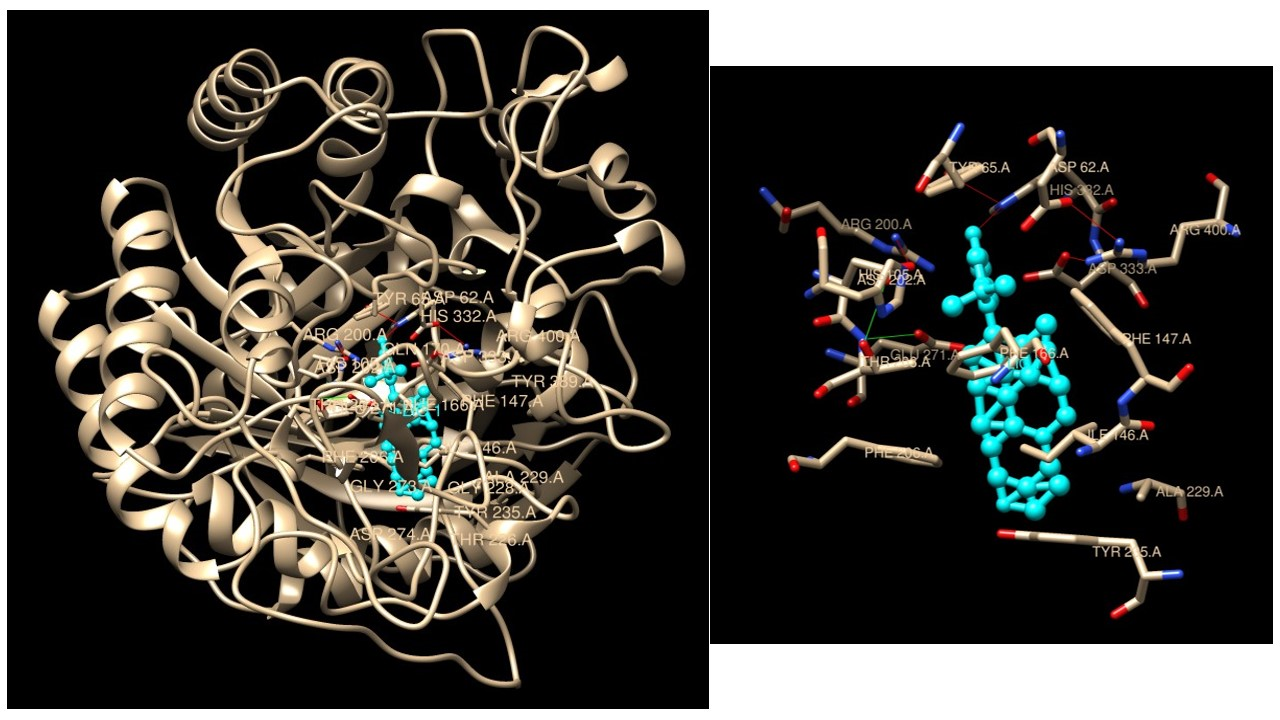


**Additional file 2: Figure S1.**


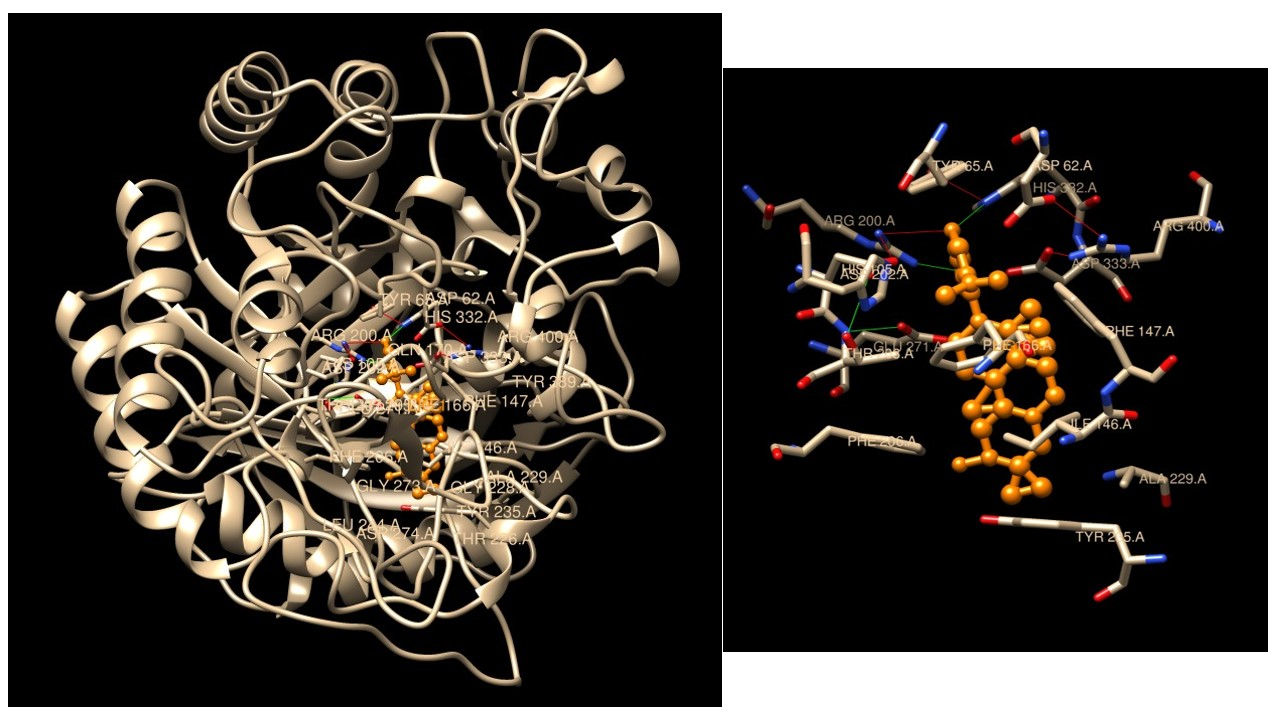


**Additional file 2: Figure S2.**


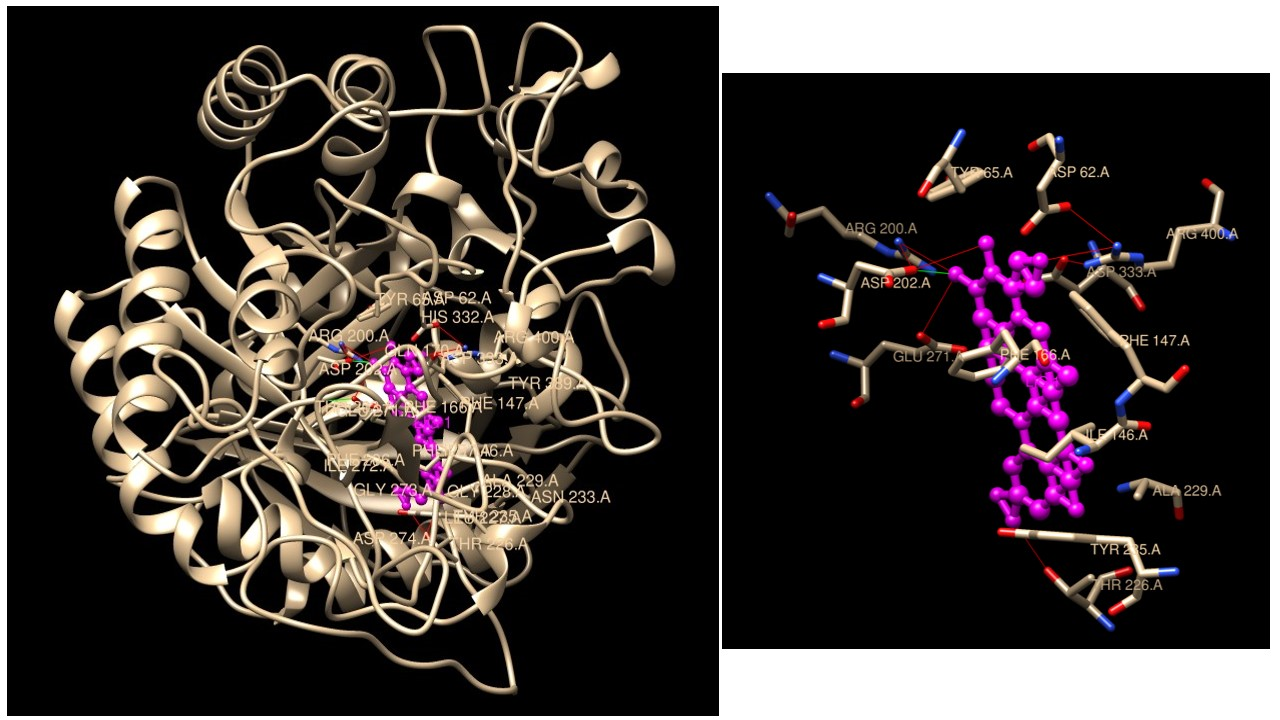


**Additional file 2: Figure S3.**


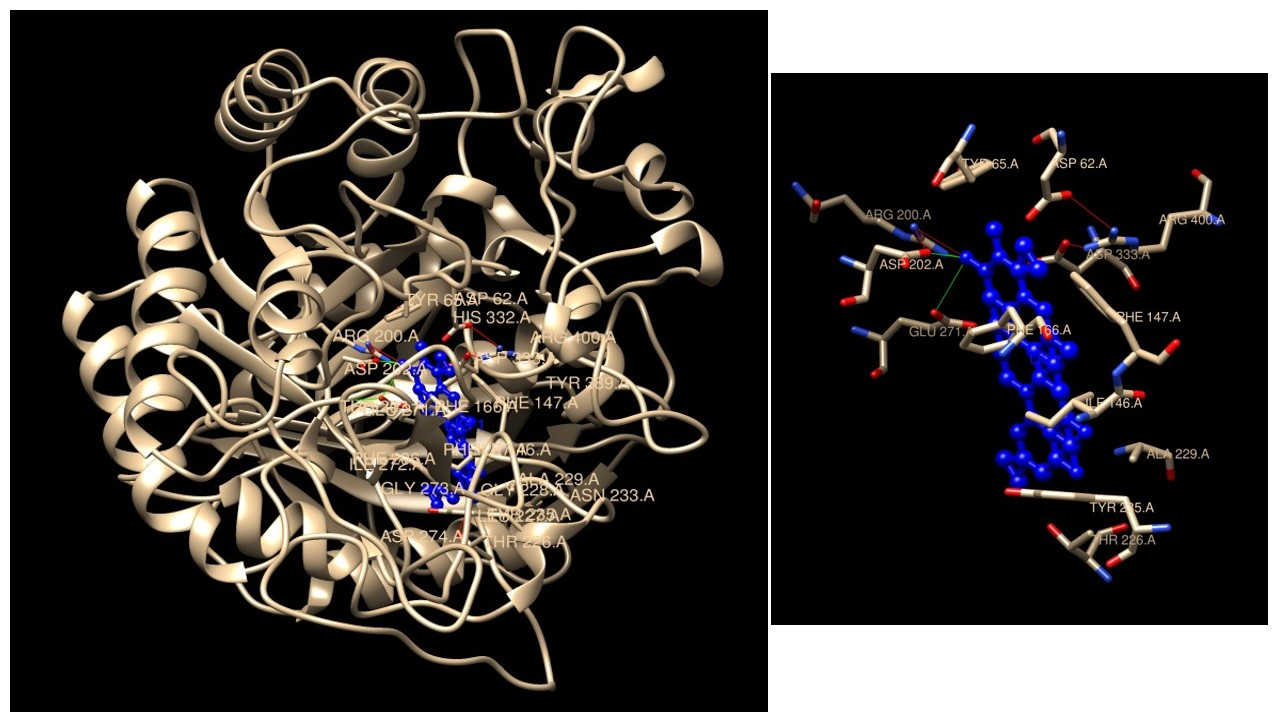


**Additional file 2: Figure S4.**


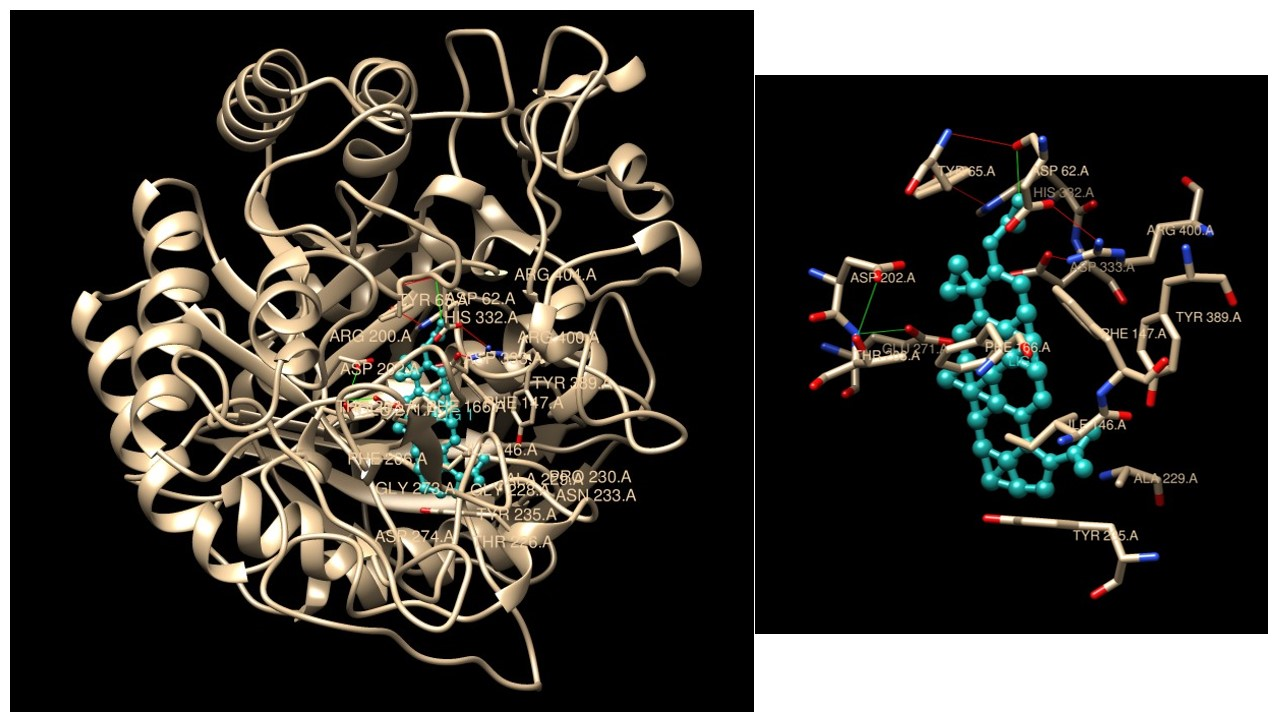


**Additional file 2: Figure S5.**


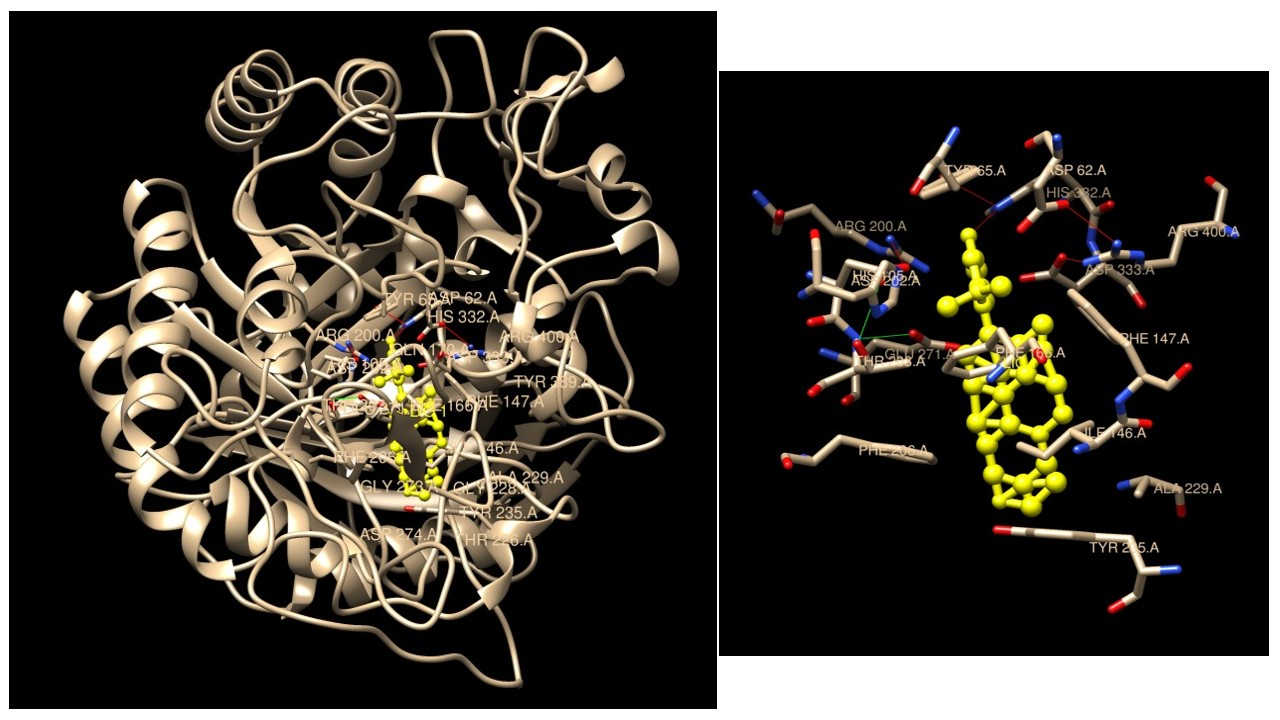


**Additional file 2: Figure S6.**


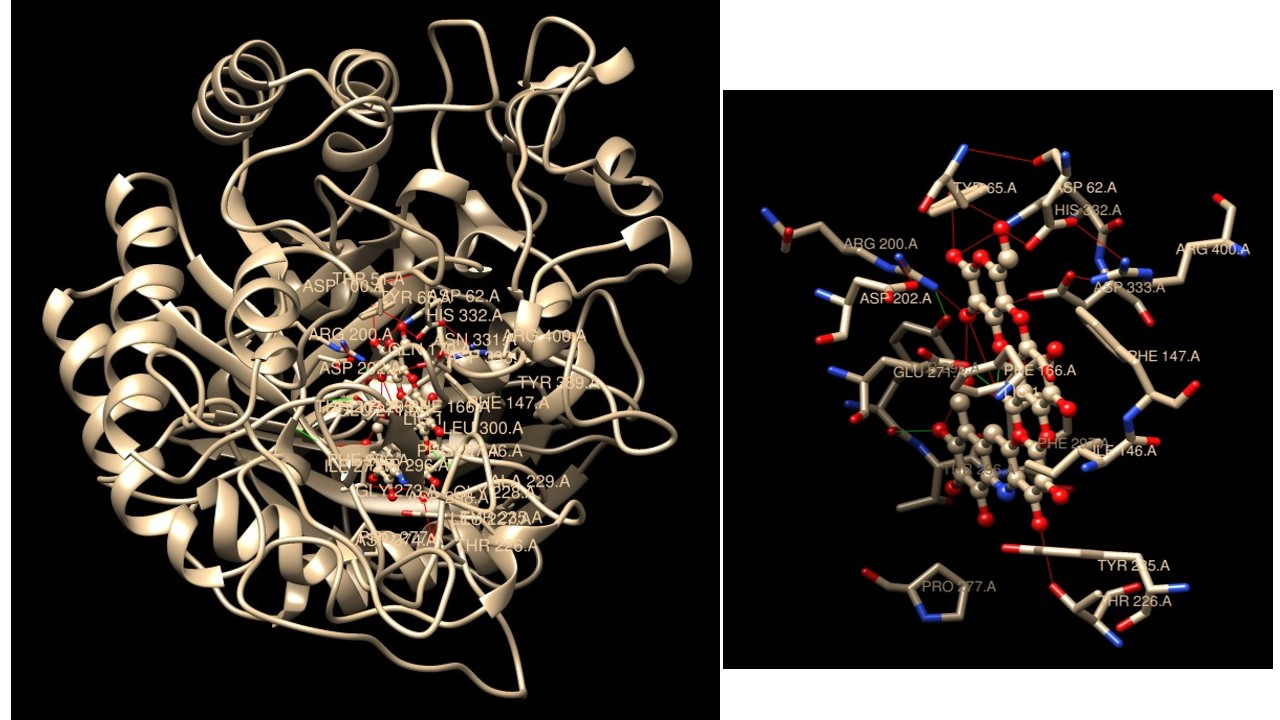


**Additional file 2: Figure S7.**





**Additional file 2: Figure S8**
